# Counteracting mental fatigue for athletes: a systematic review of the interventions

**DOI:** 10.1186/s40359-023-01476-w

**Published:** 2024-02-09

**Authors:** He Sun, Kim Geok Soh, Alireza Mohammadi, Zakaria Toumi, Lingling Zhang, Cong Ding, Xiaojuan Gao, Jian Tian

**Affiliations:** 1 School of Physical Education, Henan University, Kaifeng, China; 2 Department of Sport Studies, Faculty of Education Studies, Universiti Putra Malaysia, Serdang, Selangor Malaysia; 3 Faculty of Business Management, City University Malaysia, Selangor, Malaysia; 4 School of Psychology, Northeast Normal University, Changchun, China; 5 The National Football Academy, Shandong Sport University, Jinan, China

**Keywords:** Motor performance, Perceptual-cognitive skill, Attention resources, Self-regulation, Intervention

## Abstract

The deleterious effects of mental fatigue (MF) on athletes have been carefully studied in various sports, such as soccer, badminton, and swimming. Even though many researchers have sought ways to ameliorate the negative impact of MF, there is still a lack of studies that review the interventions used to counteract MF among athletes. This review aims to report the current evidence exploring the effects of interventions on MF and sport-specific performance, including sport-specific motor performance and perceptual-cognitive skills. Web of Science, Scopus, PubMed, and SPORTDicus (EBSCOhost) were combed through to find relevant publications. Additionally, the references and Google Scholar were searched for any grey literature. For the current review, we included only randomized controlled trials that involved athletes, a primary task to induce MF, interventions to counter MF with comparable protocols, and the outcomes of sport-specific motor performance and perceptual-cognitive skill. The selection criteria resulted in the inclusion of 10 articles. The manipulations of autonomous self-control exertion, person-fit, nature exposure, mindfulness, and transactional direct current stimulation showed that positive interventions counteract MF and improve sport-specific performance in different domains, including strength, speed, skill, stamina, and perceptual-cognitive skills. The selected interventions could significantly counteract MF and improve subsequent sport-specific performance. Moreover, self-regulation and attention resources showed the importance of the potential mechanisms behind the relevant interventions.

**Supplementary Information:**

The online version contains supplementary material available at 10.1186/s40359-023-01476-w.

## Introduction

In recent years, MF has emerged as a factor affecting sport-specific performance, distinct from the more conventional phenomenon of “physical fatigue” [1, 2]. A psychobiological syndrome caused by extended cognitive effort, MF is characterized by sensations of “fatigue” and “loss of energy” [3, 4]. One of the initial studies found MF to have negative effects on endurance [4]. Since then, the impairment of a variety of motor performance skills has been detected in athletes, such as intermittent endurance [5] and passing skill in soccer [5, 6]; visuomotor skill in basketball [7]; goal kicking skill in Australian football [8]; and 1500-m swimming performance [9]. MF specifically impairs athletes’ capacity to sustain performance during high-intensity periods, which is critical in scenarios requiring intermittent endurance, as demonstrated by the Yo-Yo Intermittent Recovery Test [5]. This form of fatigue causes a subjective amplification of perceived exertion, making physical tasks appear more difficult than they are [5, 9, 10]. Consequently, players may experience significant difficulties exerting effort during sprints. Moreover, MF has a detrimental effect on a player’s cognitive functions, prominently reflected in prolonged reaction times and reduced decision-making efficiency [10, 11]. Such cognitive impairments can lead to decreased accuracy in both passes and shoots [6], consequently affecting the overall performance and strategic implementation within the game context.

Besides motor performance, some studies such as Fortes, Lima-Junior [12], Fortes, Lima-Junior [13], Gantois, Ferreira [14] have argued that the perceptual-cognitive skill (e.g., decision-making) is significantly influenced in MF. Specifically, perceptual-cognitive skill is the capacity to extract relevant clues and combine them with available knowledge to respond properly [15]. Accordingly, any deviation from an optimal perceptual-cognitive skill could have significant consequences for game outcomes [16].

Notably, MF induces an abnormal increase in rating perception of effort (RPE) proposed in the psychological model of exercise [17]. It indicates that MF influences the concentration of adenosine with the activation of the anterior cingulate cortex (ACC) and a corresponding decrease in dopamine [3, 18]. This proposition has been confirmed in an experiment related to sport-specific performance (e.g., intermittent stamina) [19]. Conversely, the application of transcranial direct current stimulation (tDCS) has been shown to have the ability to hinder the advancement of RPE during performance tasks (e.g., endurance) [20]. Therefore, tDCS has received scholarly attention as a potential method for reducing MF effects and improving performance in the sports context [21–23]. However, the existing literature portrays a varied array of results regarding this issue. Penna, Filho [23] found no statistically significant changes in completion time during an 800 m swimming trial after administering tDCS, whereas Nikooharf Salehi, Jaydari Fard [21] observed positive results in mitigating the negative effects of MF in a 50 m swimming task. Given the contrasting viewpoints, there is a need to conduct a comprehensive review that aims to clarify and integrate the available information regarding the effectiveness of these interventions.

In addition, a potential extension for the model is proposed by a recent study [24]. Due to the different mechanisms of the components of sport-specific performance, Sun and colleagues suggested adding a third factor of directed attention into the model to explain the impairment of perceptual-cognitive skill (e.g., decision-making). Apart from explaining the adverse effects of MF, these theories have also been used to form potential strategies to counteract MF, especially in athletes. Therefore, Sun, Soh [25] conducted a study involving an intervention utilizing virtual nature stimuli, positing that mentally fatigued athletes who engaged in a 12.50-min exposure within natural scenes exhibited a diminished decline in soccer decision-making, potentially attributable to increased attention resources (e.g., directed attention). Consequently, a particular question arises regarding the interaction between directed attention and self-regulatory resources, considering their acknowledged conceptual similarity [26]. Could manipulation of self-regulation, in effect, also serve as a strategy to mitigate the impact of MF? Is it possible that professional athletes, due to their presumably higher levels of self-regulatory resources [27], demonstrate better performance in sport-specific tasks compared to athletes at lower competitive levels?

It is important to highlight that the existing body of literature lacks a comprehensive review, resulting in an unclear understanding of the mechanism of counteractive interventions, such as the involvement of attention and self-regulatory resources. Despite the presence of specific investigations that have provided empirical evidence supporting the effectiveness of manipulations for self-regulation such as autonomy [28] and person-fit [27], this gap continues to exist.

In the most recent year, some investigations have begun to get promising results from analysing the interventions in this field. For example, Oliver, Sullivan [29] analysed nutritional interventions counteracting MF in three populations, including sporting, military, and aerospace. The result showed positive effects for MF and improvement of cognitive skills (e.g., reaction time). Consistent with the psychobiological model, Azevedo, Silva-Cavalcante [30], Franco-Alvarenga, Brietzke [31] demonstrated that caffeine intake led to a decrease in RPE under mentally fatiguing conditions and improved subsequent athletic performance (e.g., cycling endurance). However, some supplements such as caffeine could have excessive effects, such as abnormal nervousness, irritability, insomnia, and sensory disturbances [32]. Furthermore, the effects of MF are lessened only after using caffeine or a carbohydrate mouth rinse for 40 min or 15 min, respectively, and only occur after several days, such as creatine [33].

Another most recent review conducted by Proost, Habay [34] examined some strategies to counteract MF. However, only the potential countermeasures were emphasized. More importantly, they did not focus on sport-specific performance in athletes.

Therefore, the current review aims to investigate all the evidence on different interventions for reducing the impact of MF and improving subsequent sport-specific performance, including motor performance and perceptual-cognitive skill in athletes.

## Methods

The review complies with the Preferred Reporting Items Checklist (PRISMA) requirements for reporting [35]. Four major databases (Web of Science, SPORTDicus through EBSCOhost, PubMed, and Scopus) were used to conduct a thorough search of published works from the time they were published until December 2022 (Supplementary Table S1). In addition, citations and reference lists were combed to identify more studies.

### Eligibility criteria

The PICOS method was used to look for literature (Table 1). Articles were considered if they met certain requirements: (a) considered a variety of levels of athletes (e.g., amateur, semi-professional, and professional) without any injury; (b) included one of five components of sport-specific motor performance (strength, speed, stamina, flexibility, and skill) or perceptual-cognitive skill; (c) investigate interventions aimed at mitigating MF within the intervention group and use various controls—whether passive, placebo, or wait-list—in the control group) execute any intervention to minimise MF. When employing a placebo control, the study must clearly specify the type of placebo implemented; (d) recruited a task for prior mental exertion to induce MF condition; (e) published the results with a randomized controlled trial; and (f) peer-reviewed articles in English.

**Table 1:** PICOS criteria

**PICOS**	**Criteria**
Participation	Athlete, with no restrictions on their sport activity, gender, or age
Intervention	Manipulations to counteract MF whilst measuring subsequent sport-specific performance without any supplements
Comparison	Intervention vs. non-intervention groups (e.g., passive, placebo, or wait-list control group)
Outcome	Sport-specific motor performance and perceptual-cognitive skill
Study Design	Randomized Controlled Trial

Notably, in the current review, skill is defined as the capacity to perform tasks at a high level while also being effective and efficient [36]. It is alternatively known as technical performance or skilful sports execution [2, 37]. In ball games like basketball and soccer, skill refers to the player’s ability to control the ball. This includes shooting, passing, tackling, and dribbling the ball in a way that helps the team.

The incorporation of perceptual-cognitive skills in the current analysis holds significant practical significance, as it enables athletes to perceive and understand complex patterns within the competitive environment (e.g., opponents’ actions and behaviours). Consequently, perceptual-cognitive skill acts as a catalyst for prompt reactions, resulting in the implementation of motor execution aimed at achieving optimal performance [38].

### Literature search and selection

**Fig. 1:**
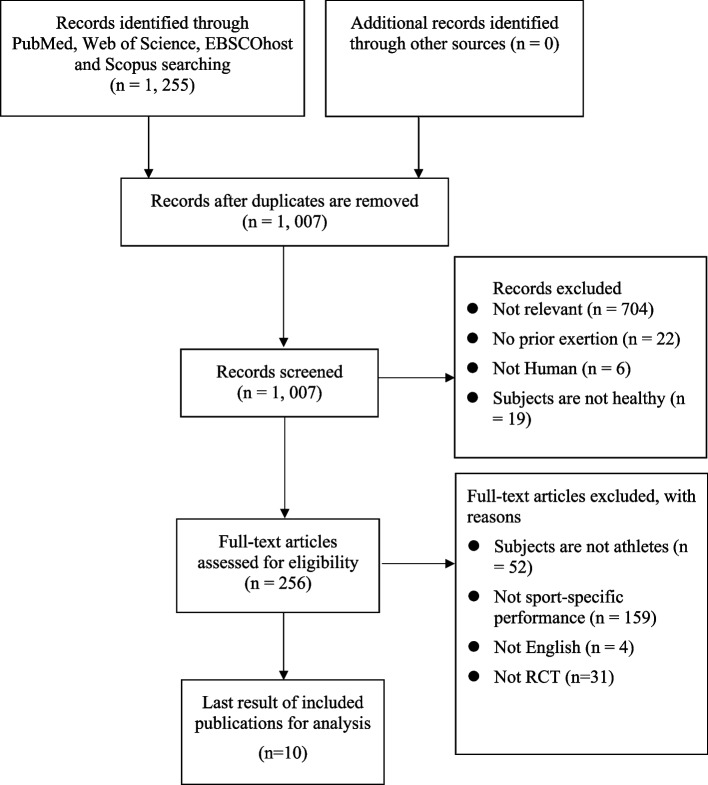
PRISMA summary of selection procedure

The databases were searched using the keywords, truncation, and Boolean operators shown in Supplementary Table S1. Additional material was searched through references and Google Scholar. Two independent reviewers examined the article abstracts, titles, and search results to find articles that satisfied the requirements. A full-text evaluation of 256 papers followed the screening (Fig. 1). In addition, a third reviewer was consulted when disagreements arose.

### Protocol and registration

The protocol used for methodology and planned analysis was recorded in OSF Registries (https://osf.io/9nz26). Thus far, no protocols have examined the impact of non-supplement interventions on sport-specific motor performance and perceptual-cognitive competence simultaneously. Therefore, the suggested protocol’s originality is ensured.

### Risk of bias assessment

The risk of bias in various studies was evaluated using the Revised Cochrane Risk of Bias instrument for randomized trials (RoB 2.0). “Low risk of bias,” “high risk of bias,” or “some worries of bias” were assigned to each of the following five categories based on signalling questions. Reviewers followed the guidelines set by Cochrane.

## Results

### Literature selection

The initial phase of searching yielded a total of 1255 unique studies. After carefully removing any duplicates, a collection of 1007 research papers were selected for further examination. After conducting a thorough examination of the titles and abstracts, a total of 751 studies were found to be inconsistent with the research objectives and were subsequently eliminated from further evaluation. The third phase involved a comprehensive assessment of 256 full-text papers conducted by two independent reviewers, both of whom agreed on the eligibility of the included studies. After a thorough and meticulous evaluation, there were 10 research studies that met all the specified qualifying criteria. Therefore, these 10 research studies were included in the current review. The visual representation of the selection process is illustrated in Fig. 1.

### Risk of bias

**Fig. 2:**
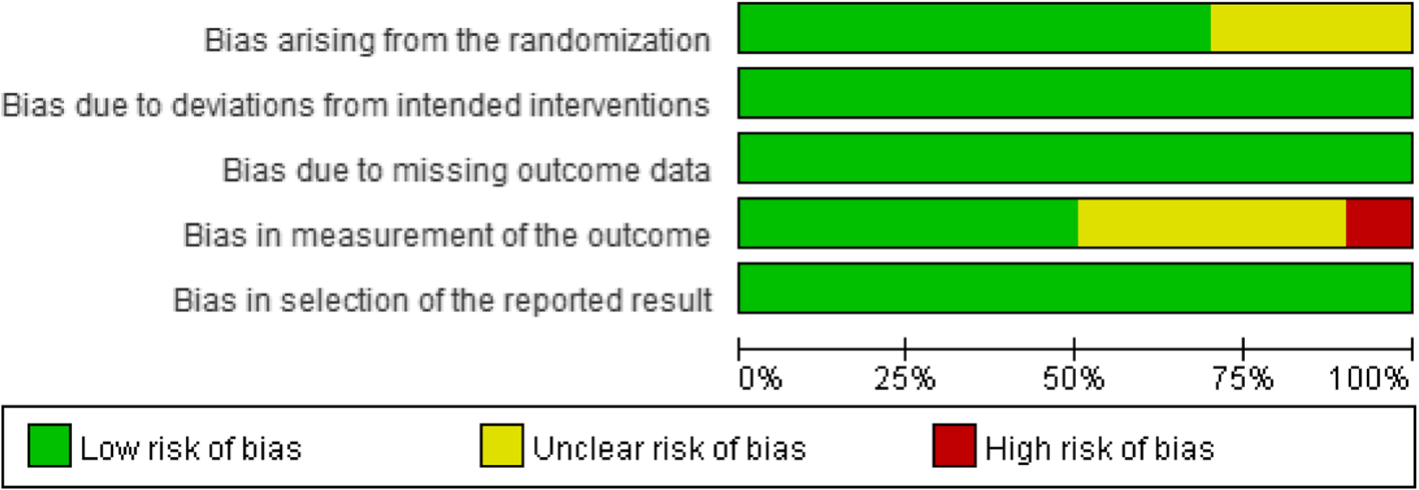
Risk of bias for all included studies

**Fig. 3:**
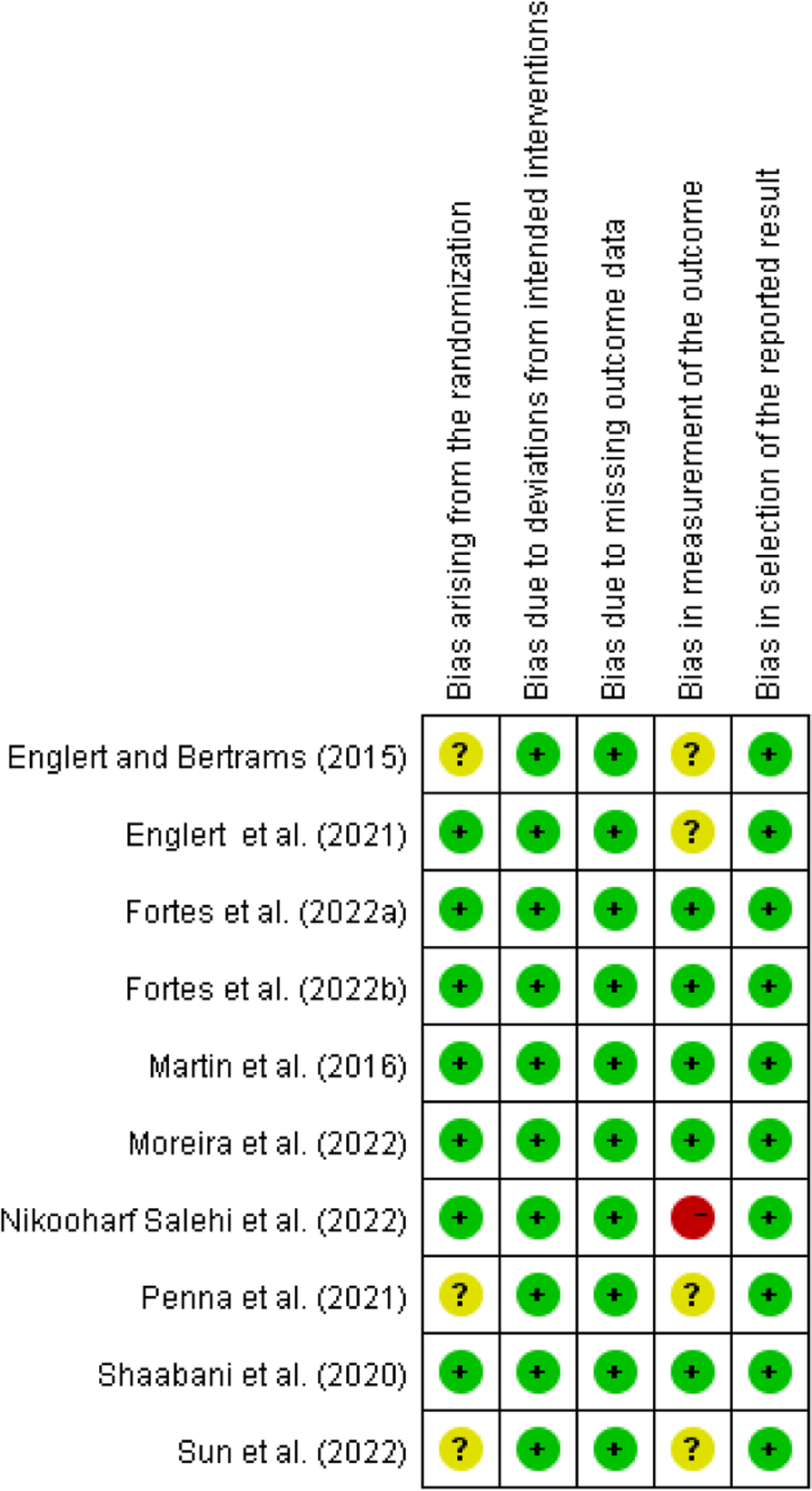
Risk of bias summary for each included study

One study [21] had a high-risk bias in the outcome due to unblinding of the assessors. The other four studies were considered to have an “unclear risk of bias” for unclear evidence. Three studies [23, 25, 28] were rated as having some concerns about bias due to no information on allocation concealment. The details are shown in Figs. 2 and 3.

### Population characteristics

A total of 316 participants were included (Table 2). The ratio of female to male participants was 279:37. The average age was 19.4 [39] to 30.0 years [23]. The professional level of athletes comprised the primary population.

**Table 2:** Overview of included publication details

**NO.**	**Reference**	**Population Characteristics**	**Intervention**	**Prior Mental Exertion**	**Duration of the Prior Mental Exertion**	**Main Outcome**		**Psychophysiological Outcome**	**The domain of the Outcome**	**Sports**
1	Englert and Bertrams [28]	57 semi-professionals Sex: 29 ♂; 28 ♀ Age: 24.7 ± 4.5	Autonomous self-control exertion	Transcription task	5 min	Serve accuracy↑		Sports anxiety ↔	Skill	tennis
2	Martin, Staiano [27]	11 professionals; 9 recreational cyclists Sex: 20♂ Age: 23.4 ± 6.4	Person-fit	Stroop task	30 min	Cycling Power in professionals↑		RPE ↔ HR ↔ Blood lactate ↔	Strength Speed	cycling
3	Shaabani, Naderi [40]	72 well-trained athletes Sex: 72 ♂ Age: 28.6 ± 4.0	Mindfulness	Stroop task	15 min	Shooting accuracy↑		Sport anxiety ↔ Depletion sensitivity ↔ Positive and negative affective states ↔	Skill	basketball
4	Englert, Dziuba [39]	23 elites Sex: 12♂; 11♀ Age: 19.4 ± 4.1	Person-fit	Transcription task	5 min	Shooting accuracy↑		perceived self-control strength ↔	Skill	shooting
5	Penna, Filho [23]	10 elites Sex: 10♂; Age: 30.0 ± 6.0	tDCS	Stroop task	45 min	800 m swimming trial ↔		Motivation ↔ RPE ↔	Stamina	swimming
6	Sun, Soh [25]	90 university athletes Sex: 90 ♂ Age: 20.7 ± 2.0	Nature exposure	Stroop task	30 min	Soccer decision-making↑		Motivation ↔ RPE ↔	Perceptual-cognitive skill	soccer
7	Nikooharf Salehi, Jaydari Fard [21]	15 professionals Sex: 15♀ Age: 23.0 ± 1.0	tDCS	Stroop task	60 min	50 m swimming trial↑			Speed	swimming
8	Fortes, Ferreira [41]	20 professionals Sex: 20♂ Age: 24.8 ± 4.2	tDCS	Sport-based videogame	60 min	Basketball decision-making↑ Visuomotor skill↑	Eyeblink duration ↔ Pupil diameter ↔ Subjective MF ↓ Motivation ↔		Perceptual-cognitive skill	basketball
9	Moreira, Moscaleski [42]	9 professionals Sex: 9♀ Age: 25.0 ± 8.0	tDCS	Stroop task	30 min	Shooting accuracy ↔	RPE ↔ Success motivation ↔ Intrinsic motivation ↔ Subjective workload ↔		Skill	basketball
10	Fortes, Faro [22]	19 amateur athletes Sex: 19 ♀ Age: 20.2 ± 1.5	tDCS	Stroop task	30 min	Tethered swimming↑	Subjective MF ↓ Motivation ↔		Strength	swimming

*RPE* Rating perception of effort, *HR* Heart rate, *tDCS* Transcranial direct current stimulation, *MF* Mental fatigue

Since the perceptual-cognitive skill is especially challenging in open-skill sports [16], we divided all sports into two categories: open-skill sports, such as tennis [28], basketball [40–42], and soccer [25]; and closed-skill sports, such as cycling [27], shooting [39], and swimming [21–23].

### The counteractive effects of the investigated interventions on sport-specific performance

#### The intervention of autonomy-supportive environment

The autonomy-supportive environment refers to a situation that enables athletes to satisfy their basic needs for autonomous acting and decision-making to lead to better performance in sports [28, 43]. Englert and Bertrams [28] initially investigated a particular intervention that focused on the creation of an autonomy-supportive environment. This environment was designed to aid participants in making decisions regarding whether or not to cease their efforts in a preceding MF task. Specifically, they examined the negative impact on tennis serve skill under conditions of high pressure. The results showed that the autonomy-supportive group significantly outperformed the control group (no autonomy-supportive manipulation) in serving accuracy (M = 17.10, SD = 6.43 vs. M = 13.05, SD = 3.99; *p* = 0.04) with a prior transcription task.

#### The intervention of person-fit

Two separate investigations have been conducted to examine the intervention of person-fit. Martin, Staiano [27] examined the strength of cycling performance and revealed that professional athletes with better person-fit could resist MF and maintain power generated on a cycle ergometer. Specifically, professional cyclists had no significant main impacts on the condition (mental fatigue vs. non-MF: *p* = 0.675, ${\upeta }_{p}^{2}$ = 0.020), and recreational athletes’ power dropped significantly (*p* = 0.017, ${\upeta }_{p}^{2}$ = 0.530) [27]. Similarly, in the sport of shooting, shooting accuracy did not significantly drop in elite-level (better person-fit) athletes after a MF task compared with the non-MF group (*p* > 0.05) [39]. Moreover, Martin, Staiano [27] also found that professional cyclists could maintain average speeds well. In other words, the average speed was not considerably different (44.1 ± 2.2 vs. 44.3 ± 1.8, *p* = 0.261, ${\upeta }_{p}^{2}$ = 0.138) in the time trial.

#### The intervention of nature exposure and mindfulness

Exposure to nature and the practice of mindfulness have been examined as effective interventions to counter MF and enhance subsequent sport-specific performance. Specifically, Sun, Soh [25] examined the effect of virtual nature as an intervention and found that a 12.50 min intervention could significantly improve soccer decision-making skills in reaction time (M = 5.01, SD = 1.46 vs. M = 7.21, SD = 1.65; *p* < 0.01; ${\upeta }^{2}$ = 0.08), whereas there was no significant difference in accuracy (M = 69.13 SD = 4.78 vs. M = 66.87 SD = 4.81; *p* = 0.91; ${\upeta }^{2}$ = 0.02).

Shaabani, Naderi [40] performed a study that implemented a brief mindfulness intervention consisting of a 15-min breath and body mindfulness audio exercise. In contrast, the control group participants (no mindfulness) listened to an audiobook. The results showed that basketball shooting accuracy across 30 free throws was significantly higher in the depleted group following the brief mindfulness exercise, as compared to the no-intervention group (M = 49.39, SD = 8.32 vs. M = 40.73, SD = 8.72; *p* < 0.05) [40].

#### The intervention of transcranial direct current stimulation

The majority of investigations (five out of ten studies) have converged on the use of transcranial direct current stimulation (tDCS). This is likely due to its characteristics as a non-invasive form of brain stimulation that can increase cortical excitability [44, 45]. However, different studies have produced varying results.

Specifically, Fortes, Faro [22] applied tDCS to the orbital prefrontal cortex and detected a significant main effect of condition on the fatigue index (*F* = 6.51; *p* = 0.04; d = 0.34). This resulted in the maintenance of mean force and critical force in the tDCS group, while a significant drop was observed in the sham simulation group. Similarly, when applied to the midtemporal area, tDCS was found to be effective in decreasing MF (*P* < 0.05) compared with the sham group. The improved condition was associated with enhanced basketball visuomotor and decision-making skills as measured by reaction time and accuracy [41]. Nikooharf Salehi, Jaydari Fard [21] found that tDCS could significantly reduce the negative effect of MF and improve swimming speed at 50 m compared with the sham stimulation group (25.93 ± 1.32 s vs. 27.27 ± 1.68 s; *p* ≤ 0.01).

However, Moreira, Moscaleski [42] did not detect the effects of tDCS on basketball shooting accuracy among female athletes after a 30-min Stroop task. That is, the number of shots to achieve 10 hits in undefended (M = 23.4, SD = 9.1 vs. M = 22.5, SD = 7.6; *p* = 0.651) and defended tests (M = 22.2, SD = 6.1 vs. M = 21.8, SD = 9.0; *p* = 0.681) were similar in two conditions (tDCS vs. sham – tDCS) [42]. Finally, Penna, Filho [23] showed similar performance of stamina in the comparison of the tDCS group and control group (692 ± 50 s vs. 692 ± 42 s, *p* > 0.05) during an 800 m swimming trial.

### Psychophysiological outcome

Remarkably, the psychophysiological outcomes were consistent (Table 2). The indicators of MF, such as eye blink duration, pupil diameter [41], RPE [23, 25, 27, 42], and sports anxiety [28, 40], showed no significant difference between groups following the interventions. While subjective reports of MF significantly increased after the MF task, a notable decrease was observed in the tDCS intervention group [22, 41]. This decline can be attributed to the efficacy of tDCS in countering MF.

## Discussion

We evaluated the existing literature on interventions that mitigate MF and subsequently enhance sport-specific performance, including sport-specific motor performance and perceptual-cognitive skill. Given the detrimental effects of MF, the present findings offer insights into potential interventions that can alleviate MF and improve sport-specific performance.

### Sports characteristics

The academic study of MF in sports was initiated by Smith, Marcora [19]. The authors first reported on the impairment of intermittent endurance among various types of athletes (e.g., team sports). Since then, the effect of MF has been examined comprehensively in many sports in recent years. However, studies on interventions for MF have been limited to six sports (Table 2). As MF affects sport-specific performance, more studies in other sports are required in the future, such as boxing [11], table tennis [46], cricket [47], and Australian football [8].

Notably, some specific characteristics of the sports make them relevant subjects for such studies. For example, sports like tennis, basketball, and soccer involve a lot of tactical awareness, making decisions under pressure, and team coordination [48, 49]. MF directly impacts these cognitive aspects. In addition, MF influences pacing [19] and self-selected power output [50] in endurance sports like swimming and cycling. Finally, shooting is a sport that demands extreme concentration and precision. Even minor lapses due to mental fatigue in attention can significantly affect performance [51].

Moreover, there are only two studies that examined perceptual-cognitive skills in soccer and basketball. The skills refer to the abilities that allow athletes to process and interpret visuals rapidly and accurately. These skills are crucial for recognizing patterns, making quick decisions, anticipating opponents’ actions, and more. In the context of sports, these skills enable athletes to respond effectively to dynamic and often unpredictable game situations [52]. In the current review, the visuomotor [41] and decision-making skills [25, 41] were examined. On one level, it is the main determining factor as to whether athletes will function well at superior levels (more rapid and accurate) [53]; on another level, it could determine competition results [16]. Future studies should examine these skills in more detail, especially in open-skill sports due to the dynamic and complex competitive environment.

### Interventions characteristics

In the current review, a variety of intervention types have been found to have a counteractive effect on MF and improve the subsequent sport-specific performance, including sport-specific motor and perceptual-cognitive skills. These interventions are discussed in the sections below.

#### The counteractive effect of autonomy-supportive environment

The autonomy-supportive environment is one in which coaches cultivate a milieu that positively encourages athletes to exhibit initiative and engage in self-directed decision-making processes [54, 55]. This approach contrasts with a controlled environment in which decision-making is mostly centralised in coaches, severely limiting athletes’ sense of autonomy. Empirical evidence has demonstrated a role of autonomy support. Specifically, athletes experiencing a greater degree of autonomy are more likely to persevere, nurture creative ideas within their sporting disciplines, and accomplish significant advances in skill development [56, 57].

In such an environment, athletes demonstrate a tendency for increased active participation in training sessions and competitive events [58, 59]. This enhanced involvement is characterized by a propensity to independently initiate personal development, actively seek out challenges, and demonstrate a higher level of commitment in both practice and competitive settings. Moreover, the environment could be conducive to the development of more positive relationships between coaches and athletes, characterized by mutual respect and a deeper mutual understanding [60].

In the study encompassed within this review, Englert and Bertrams [28] found that the autonomy-supportive environment (e.g., athletes have the right to determine whether or not to exert self-regulation) could attenuate the detrimental effects of MF on second serve in tennis under high-pressure conditions.

The effect could be explained by two theories, namely, the self-determination theory and the resources model of self-regulation. Specifically, the resources model indicates that self-regulation is a limited “reservoir”. It is seen as limited over time, along with physical and cognitive performance that requires self-regulation [61, 62]. On the other hand, it can also be exercised and increased significantly [63]. In a recent extensive analysis conducted by Sun, Soh [64], it was found that training programmes incorporating self-regulatory strength have a beneficial impact on MF and subsequent physical and cognitive performance. This finding aligns with the resources model of self-regulation, as posited by the resources model of self-regulation. To date, only one study has been undertaken on athletes [28], indicating a promising area for future research.

Moreover, the self-determination theory provides a framework to comprehend how these states of autonomy (or a rather widespread effect sensation of being compelled to act) lead to varied practical results [65, 66]. At its foundation, the self-determination theory proposes two types of motivation: intrinsic motivation refers to doing something out of interest or delight, whereas extrinsic motivation refers to doing something for instrumental purposes. Notably, situations with autonomy-supportive encouragement could increase levels of intrinsic motivation among athletes. Therefore, athletes with an autonomy-supportive environment can be inspired to put forth greater effort using more resources in the “reservoir” and show higher performance even under MF (e.g., [28]: tennis serving skill).

Given that Englert and his colleague also conducted their study under conditions of high pressure, such environments may be instrumental in reducing stress among athletes, fostering a sense of control pressure in their sporting activities [67].

#### The counteractive effect of person-fit

Person-fit in the sports context is explained as the compatibility between an athlete’s characteristics and those of a specific sport-specific task [68]. Undoubtedly, athletes who have higher competitive levels, such as professionals and elites, possess better person-fit [69]. With regards to lifestyle, high-level athletes (e.g., professional and elite) are more likely to be in situations that require self-regulation and inhibition control more often than low-level athletes (e.g., recreational and non-elite) [70, 71]. High-level athletes must regulate their nutrition and alcohol consumption, refrain from smoking, ensure adequate rest, and adhere to a rigorous physical training regimen. This persistent self-regulation of behaviour may increase inhibitory control throughout the physical and cognitive domains [27, 72]. For example, a recent systematic review reported that individuals who spent several weeks doing self-regulatory exercises (posture regulation, financial monitoring, and non-dominant hand use) performed better in physical and cognitive tasks following prior MF task [64].

In the current review, better person-fit or high-level athletes (e.g., professional or elite) showed a superior ability to attenuate the negative impact of MF and maintain other sport-specific performance such as cycling strength and speed [27] and shooting accuracy [39], compared with their counterparts (e.g., recreational and non-elite). High-level athletes might nonetheless experience MF. For example, professional athletes showed worse soccer decision-making after a 30-min use of social networks on smartphones or playing video games [73]. Future studies can investigate this discrepancy by using the same mentally fatiguing task with the same duration.

#### The counteractive effect of nature exposure and mindfulness

Nature exposure and mindfulness meditation are two promising interventions. Long-time (e.g., 12.50 min) exposure to nature scenes significantly improved soccer decision-making skills due to directed attention as well as self-regulation restoration [24, 25]. Since there is an overlap between directed attention and self-regulation [26], Sun and colleagues further proposed a conceptual framework to show the mechanism of the improvement regarding self-regulatory capability and perceptual-cognitive skills such as decision-making [24]. However, the proposition of the conceptual model should be tested through more empirical studies in the sport context. For example, competitive state anxiety and heart rate variability can be tested directly after the intervention of nature exposure, since they could be indicators of self-regulation [74, 75].

Self-regulation and mindfulness are linked by research [76, 77] as they share some common mechanisms. Notably, Friese, Messner [78] indicated that mindfulness meditation attenuates the depleted effect through the restoration of attention. Moreover, Bishop, Lau [79] emphasized the importance of self-control of attention as a component of mindfulness. Several questions were prompted. For example, could attention be a common resource for self-regulation and mindfulness? Perhaps, mindfulness meditation also could be manipulated for attention and integrated into the conceptual framework [24]. Moreover, since there was a threshold for the intervention of nature exposure to counteract mental fatigue and improve soccer decision-making [25], is there also a threshold for the intervention of mindfulness to restore attention resources? Nevertheless, it is necessary to examine this more deeply in future studies.

#### The counteractive effect of transcranial direct current stimulation

In recent years, five research studies have examined tDCS as an ergogenic aid to combat mental tiredness in the athlete population (see Table 2). The technique, which involves delivering a small electrical current to the scalp to raise (anodal tDCS: a-tDCS) or decrease (cathodal tDCS: c-tDCS) neuronal excitability for sustained durations [80], has been demonstrated to reduce the aberrant rise in RPE [81, 82], a phenomenon linked to the impairment of sport-specific performance as per the psychobiological model [47, 83]. The potential mechanism underlying the ergogenic effect of preventing MF may be the increased cortical excitability in certain brain regions targeted by anodal stimulation, such as the left temporal cortex [23], the middle temporal area [41], the dorsolateral prefrontal cortex [21, 42], and the orbital prefrontal cortex [22].

Although tDCS was anticipated to have a widespread effect [84], it did not improve cognitive performance in MF and subsequent swimming stamina (800 m swimming) in a study by Penna, Filho [23] (e.g., Stroop task). Thus, more research investigating the unique high-definition tDCS approach is required [85].

Consistent with the person-fit intervention, Penna et al. [23] demonstrated that the competitive state of the athletes nullified any potential favourable benefits of tDCS. Specifically, temporal brain activity is linked to the regulation of cardiac autonomic function [20, 86], and RPE is involved in this relationship [87]. However, this modulation may not have been significant enough to indicate improved performance among elite athletes (e.g., professionals). Penna and colleagues chose a group of athletes with 14 years of consistent training, and these athletes may have had enhanced temporal cortex function as a result of regular exercise.

In contrast, the other four studies that investigated tDCS showed significant improvement in the condition of MF. It increased cognitive performance (e.g., reaction time) in the mentally fatiguing task (e.g., Stroop task or video game) and in subsequent sport-specific performance such as 50 m swimming trial [21], basketball decision-making and visuomotor skill [41], basketball shooting accuracy [42], tethered swimming measured as a critical force, aerobic impulse, and mean force [22].

Notably, tDCS emerges as a potentially promising intervention for practical application. The intervention has the potential to ameliorate MF, subsequently enhancing various domains of sport-specific performance, including speed, skill, strength, and perceptual-cognitive skill, as delineated in Table 2. Perhaps more importantly, it can be applied after a mentally fatiguing task. That means there is a large possibility of applying the intervention before athletic competitions. However, more studies are required to examine different sports, such as Australian football, cricket, and table tennis, especially because their sport-specific performance is also influenced by MF [8, 46, 47].

### The potential mechanism of applied interventions

In the current review, the potential mechanism of applied interventions could be explained through the psychophysiological outcomes in Table 2. MF was significantly improved, measured as subjective and physiological indicators after the tDCS [22, 41]. The intervention probably improved attention resources and thus reduced MF as proposed by the most recent study [24]. It has been suggested by Andrew McKinley [88] that the characteristics of tDCS, such as electrode montage, duration, and intensity, are equivalent to enhancing focus and decreasing mental weariness. Both processes may share the same underlying mechanism.

Moreover, according to the resource model of self-regulation shown in the Discussion section, all the interventions investigated in the current review are related to self-regulatory capabilities among athletes. Some included studies manipulated attention resources through the intervention and showed significant results [21, 22, 25]. The resources of self-regulation and top-down attention (e.g., directed attention) are overlapping [26]. Therefore, as shown by Sun, Soh [24], the intermediate mechanisms to counteract MF and improve subsequent sport-specific performance might be self-regulation and directed attention.

In line with the psychobiological model, the traditional indicators of fatigue (e.g., heart rate and blood lactate) were not significantly different between the intervention and the control conditions [27]. Additionally, RPE as a primary component in the model was at a similar level between groups after the intervention [23, 25, 42]. This suggests that subsequent research on the intervention could be guided by the psychobiological model of exercise performance.

## Limitations

Despite being carefully conducted, the current review has a few limitations. First, a meta-analysis was not conducted due to the heterogeneity across the measurement and interventions. Second, regarding the intervention of tDCS, only a-tDCS was investigated. The effects of different types of tDCS (a-tDCS vs. c-tDCS) were not evaluated due to the limited investigations of c-tDCS, as only one study examined c-tDCS [42]. Additionally, although this review implies that high-level athletes, whether professional or elite, may exhibit heightened self-regulation capabilities that enable them to sustain their sports performance, it is important to note that they are not immune to MF. For instance, elite cricketers’ performances have been observed to deteriorate due to MF [47, 89]. However, this review could not identify interventions other than tDCS, given the constraints in recruiting professional athletes for the studies reviewed. Finally, only publications written in English were selected, which may have limited the results.

## Conclusion

A careful selection of interventions could significantly counteract MF and improve the subsequent sport-specific performance in different domains. Self-regulation and attention resources appear to be important mechanisms behind this counteractive effect. Athletes who are in an environment that encourages autonomy may feel motivated to put in extra effort by utilizing additional resources. Therefore, it is probable that they will exhibit higher levels of performance even when the MF condition is present. Another promising intervention is tDCS; however, different types of tDCS, such as a-tDCS and c-tDCS, should be further investigated in future studies.

### Supplementary Information

**Additional file 1: Supplementary Table S1.** Detailed search strategy.

## Data Availability

The datasets generated during and/or analyzed during the current study are available from the corresponding author upon reasonable request.
