# Supplementary material for: Counteracting mental fatigue for athletes: a systematic review of the interventions
Source: BMC Psychol. 2024 Feb 9;12:67. doi: 10.1186/s40359-023-01476-w (PMC10854164; doi:10.1186/s40359-023-01476-w)
Supplement: Supplementary file 1 — Additional file 1: Supplementary Table S1. Detailed search strategy. [file 40359_2023_1476_MOESM1_ESM.docx]

**Supplementary Table S1**

**Detailed search strategy**

**Search Date: 30 December 2022**

|  | Database Complete search string | Results |
| --- | --- | --- |
| PubMed | (("Athletes"[Mesh]) OR ("Athletes") OR ("Player") OR ("Sports"[Mesh]) OR ("Sport*")) AND (("Mental Fatigue"[Mesh]) OR ("Mental Fatigue") OR ("Cognitive Fatigue") OR ("Cognitive Exertion") OR ("Mental Exertion") OR ("ego depletion")) AND (("Counter*") OR ("Interven*") OR ("Recover*") OR ("nature exposure") OR ("napping") OR ("task switching") OR ("Exercise"[Mesh]) OR ("Exercise") OR ("Music"[Mesh]) OR ("Music") OR ("Binaural beats") OR (Mental Processes[Mesh]) OR ("Mindfulness") OR ("Motivation") OR ("Transcranial") OR ("Brain Stimulation")) NOT (("Multiple Sclerosis"[Mesh]) OR ("Neoplasms"[Mesh]) OR ("Stroke"[Mesh]) OR ("Disease"[Mesh]) OR ("Infections"[Mesh]) OR ("Immune System Diseases"[Mesh]) OR ("Endocrine System Diseases"[Mesh]) OR ("Digestive System Diseases"[Mesh]) OR ("Animal Diseases"[Mesh]) OR ("Respiratory Tract Diseases"[Mesh]) OR ("Patients") OR ("Burnout") OR ("Parkinson")) | 65 |
| Web of Science | TS = ((("Athletes") OR ("Player") OR ("Sport*")) AND (("Mental Fatigue") OR ("Cognitive Fatigue") OR ("Cognitive Exertion") OR ("Mental Exertion") OR ("ego depletion")) AND (("Counter*") OR ("Interven*") OR ("Recover*") OR ("nature exposure") OR ("napping") OR ("task switching") OR ("Exercise") OR ("Music") OR ("Binaural beats") OR ("Mindfulness") OR ("Motivation") OR ("Transcranial") OR ("Brain Stimulation")) NOT (("Patients") OR ("Burnout") OR ("Parkinson"))) | 179 |
| EBSCOhost  (SPORTDicus) | (("Athletes") OR ("Player") OR ("Sport*")) AND (("Mental Fatigue") OR ("Cognitive Fatigue") OR ("Cognitive Exertion") OR ("Mental Exertion") OR ("ego depletion")) AND (("Counter*") OR ("Interven*") OR ("Recover*") OR ("nature exposure") OR ("napping") OR ("task switching") OR ("Exercise") OR ("Music") OR ("Binaural beats") OR ("Mindfulness") OR ("Motivation") OR ("Transcranial") OR ("Brain Stimulation")) NOT (("Patients") OR ("Burnout") OR ("Parkinson")) | 849 |
| Scopus | (("Athletes") OR ("Player") OR ("Sport*")) AND (("Mental Fatigue") OR ("Cognitive Fatigue") OR ("Cognitive Exertion") OR ("Mental Exertion") OR ("ego depletion")) AND (("Counter*") OR ("Interven*") OR ("Recover*") OR ("nature exposure") OR ("napping") OR ("task switching") OR ("Exercise") OR ("Music") OR ("Binaural beats") OR ("Mindfulness") OR ("Motivation") OR ("Transcranial") OR ("Brain Stimulation")) NOT (("Patients") OR ("Burnout") OR ("Parkinson")) | 162 |
| Total |  | 1, 255 |
